# Construction of bicistronic cassette for co-expressing hepatitis B surface antigen and mouse granulocyte-macrophage colony stimulating factor as adjuvant in tobacco plant

**DOI:** 10.1080/13880209.2019.1662458

**Published:** 2019-09-24

**Authors:** Sara Mohammadzadeh, Hamideh Ofoghi, Mina Ebrahimi-Rad, Parastoo Ehsani

**Affiliations:** a Medical Biology Research Center, Kermanshah University of Medical Sciences, Kermanshah, Iran;; b Department of Biotechnology, Iranian Research Organization for Science and Technology (IROST), Tehran, Iran;; c Biochemistry Department, Pasteur Institute of Iran, Tehran, Iran;; d Molecular Biology Department, Pasteur Institute of Iran, Tehran, Iran

**Keywords:** Vaccine, plant-produced, GM-CSF, HBsAg, co-expression

## Abstract

**Context:** The co-delivery of adjuvant and antigen has shown to be more effective for targeting the immune response than antigen alone. Therefore, designing an efficient bicistronic system is more assuring for production of both elements in the same tobacco cells as a plant model system.

**Objective:** Comparing the efficient transient co-expression of hepatitis B surface antigen (HBsAg) and mouse granulocyte macrophage colony stimulating factor (mGM-CSF) in tobacco leaves by designing either mono or bicistronic cassettes.

**Materials and methods:** Four expression cassettes containing tobacco etch virus (TEV) leader sequence were constructed with and without above genes in different orders. The cassettes were transferred into tobacco, *Nicotiana tabacum* L. (Solanaceae), leaves by agroinfiltration technique. The expression levels were compared using ELISA and western blotting and bioactivity of cytokine was assessed by *in vitro* proliferation of mouse GM-CSF-responsive progenitor cells.

**Results:** Agroinfiltrated leaves contained recombinant HBsAg protein at 20–50 ng/mg and mGM-CSF at 0.2–4 ng/mg in both nonglycosylated and glycosylated forms. The highest expression obtained in HBsAg and mGM-CSF monocistronic co-agroinfiltrated leaves. The expression of mGM-CSF was 1.1 and 0.2 ng/mg in two different orders of bicistronic cassettes. The growth frequency of GM progenitors was approximately 1/187 cells for standard rGM-CSF and 3.2 times less activity for the plant produced.

**Discussion and conclusions:** The recombinant mGM-CSF was produced less in bicistronic cassette than other forms; however, co-presenting of both vaccine candidate and adjuvant is confirmed and could be promising for amelioration of plant expression system as a means for vaccine production.

## Introduction

Through the last decades, vaccines have been a major part of pharmaceutical proteins produced in plants to address global growing demand for the production of inexpensive and effective vaccines. The ability of plant-produced antigens to induce mucosal and systemic immune responses in laboratory animals as well as clinical trials has been confirmed by various studies (Margolin et al. 2018). Several virus-based vaccine candidates including hepatitis B core antigen (HBcAg) and hepatitis B surface antigen (HBsAg) were expressed in transgenic plants (Ehsani et al. 1997; Joung et al. 2016). However, there are also limitations associated with the transgenic expression, such as complexity of transforming process, time needed for regeneration of stable transformed plant cell, gene positional effects, low expression level and environmental concerns have shifted the studies to transient expression system (Kopertekh and Schiemann 2019). Transient expression systems could be attractive for their rapid expression, high yield, cost-effectiveness, no transgene release into the nature, as well as ease of scalability and monitoring during their biological manufacturing in plants (Mohammadzadeh et al. 2017; Kopertekh and Schiemann 2019). Preliminary experiments on the plant as edible vaccine have been indicated to be promising but due to low expression yield it often needs larger amount of recombinant plant material to be administrated (Criscuolo et al. 2019). Restriction of plant cells for high expression of some heterologous genes such as HBsAg has enhanced researchers to improve low expression. Furthermore, co-delivery of adjuvants is essential strategies to improve the immunogenicity of antigens and prevent induction of host immune tolerance especially in oral administration route or in persistent infections. Some adjuvants have been used through as mixture with plant produced proteins or as fusion proteins such as cholera toxin (Pniewski et al. 2018). In this regard, several cytokines as adjuvant have been considered because of safety and efficiency to promote of immune response to vaccine (Decker and Safdar 2011; Li et al. 2016).

Granulocyte macrophage colony stimulating factor (GM-CSF) is a naturally occurring growth factor and proinflammatory cytokine that is produced by a variety of cells in response to various immune or inflammatory stimuli. GM-CSF was able to significantly improve anti-HBs antibody titer when administrated with a booster dose of hepatitis B virus (HBV) vaccine in haemodialysis patients (Roozbeh et al. 2015). Combined therapy with GM-CSF can also promote immune response of HBV vaccine in HIV-infected individuals (Sasaki et al. 2003). GM-CSF increases the secretion of IL-2 and the proliferation of T cells. It is also important in upregulation of MHC class II and costimulatory molecules (Lin et al. 2010) and enhancing the activation of antigen-presenting cells and the cytoactivity of CTL and NK. Therefore, GM-CSF is able to promote the humoral and cellular immune responses to the hepatitis B vaccine. Despite contradictory results, GM-CSF is safe and well-tolerated that has shown promise to be a potential HBV vaccine adjuvant specially in individuals with lymphopenia and a defect in immunocompetent cells (Lin et al. 2010; Catherine and Piroth 2017). Furthermore, the fusion of GM-CSF with HBV-S in DNA vaccine format strengthened the immune responses of the HBV DNA vaccine both in normal and HBV-transgenic mice. The results demonstrated that GM-CSF could be used as adjuvant in prophylactic and therapeutic vaccination strategy to HBV (Qing et al. 2010). In another study, co-administration of HBsAg and GM-CSF using biodegradable hydrogel delivery system elicited greater Th cell proliferative and specific antibody responses compared to the conventional HBsAg vaccine (Chou et al. 2010).

In several studies, the production of biologically active human GM-CSF has been reported in plant cell suspensions (James et al. 2000; Shin et al. 2003; Kim et al. 2008), transgenic plants (Sardana et al. 2002) and in transient expression systems (Zhou et al. 2006; Vojta et al. 2015). Moreover, the expression of mouse GM-CSF in transgenic tobacco plants in its bioactive and glycosylated form was reported recently (Góra-Sochacka et al. 2010). However, the present study is a first to evaluate transient co-expression of mouse GM-CSF (mGM-CSF) and HBsAg in a bicistronic vector in tobacco, *Nicotiana tabacum* L. (Solanaceae), leaves.

In order to be able to co-express both antigen and costimulatory cytokine in the plant cell in non-fusion form to keep the native form of proteins a bicistronic vector was used. This vector contains tobacco etch virus (TEV) 5′-untranslated region (UTR) leader sequence: The 143 nt of 5′-leader is one of the most compact elements that directed cap independent translation in 5′ proximal position in a monocistronic mRNA (Zeenko and Gallie 2005). Previous work demonstrated that the TEV leader sequence enhances *in vivo* translation of mRNAs 8-21 fold in tobacco protoplasts (Carrington and Freed 1990). It also promotes translation of the second ORF when placed into the intercistronic region of a bicistronic construct, suggesting its internal ribosome entry site (IRES) activity. In bicistronic mRNA, TEV leader was able to enhance translation of the downstream cistron twofold of the control sequence (Niepel and Gallie 1999). One of the structural elements in TEV leader sequence is 5′-pseudoknot 1 (PK1) that is necessary to promote cap-independent translation. Mutational studies showed that interrupted stems or loops of PK 1 caused the loss of translational activity up to about 90% (Zeenko and Gallie 2005).

We cloned specific species murine GM-CSF in plant expression vector in order to characterize the biochemical and bioactivity of the cytokine. In this study, the expression of mGM-CSF was investigated in monocistronic and bicistronic expression cassettes in presence of noncoding TEV leader sequences to improve expression yield. Furthermore, the mGM-CSF was co-expressed along with HBsAg because of its adjuvant importance to improve the immunological response against HBsAg in vaccine formulation.

## Materials and methods

### Cell culture

The J774A.1 cells (ATCC no.: TIB-67; Rockville, MD, USA) were maintained in tissue flasks in complete medium RPMI-1640 (Biochrom AG, Berlin, Germany) with low content in phenol red supplemented with 2 mM l-glutamine, 10 mM Hepes, 24 mM NaHCO_3_, 50 µM 2-mercaptoethanol, 100 U/mL penicillin, 100 µg/mL streptomycin and 10% v/v heat-inactivated foetal bovine serum (FBS; Gibco, Paisley, UK). The J774A.1 cells were sub-cultured in 25 cm^2^ cell culture flasks (CellStar, Greiner Bio-one, Germany) at 37 °C with 5% CO_2_ in air. After incubation for 2 h, the flasks were washed three times with medium at 37 °C to remove the unattached cells, and the remaining attached cells were used as the macrophage monolayer. J774A.1 macrophages were detached from monolayer cultures by scraping, washed and pelleted twice at 500×*g* (Beckman GPR Centrifuge, Krefeld, Germany) for 10 min, resuspended in complete RPMI-1640 culture medium and counted in Malassez chambers. Cell suspensions at an appropriate concentration (2 × 10^5^ cells/mL) were added to 24-well culture plates (Sarstedt, Numbrecht, Germany) and allowed to adhere for 2 h at 37 °C under 5% CO_2_ atmosphere.

### Expression vector construction

**Figure 1.**
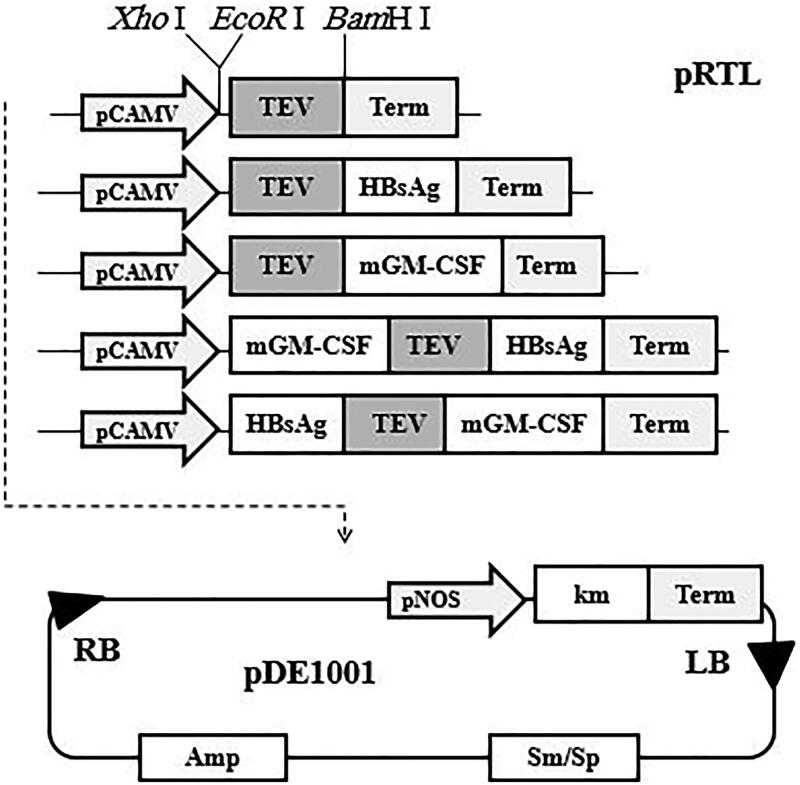
Schematic presentation of the gene arrangements in TEV-mediated bicistronic and monocistronic constructs used for co-expression of mGM-CSF and HBsAg genes.

The pRTL Plasmid (a kind gift from Dr. H. Ofoghi, IROST, Tehran, Iran) (Ofoghi et al. 2000) that carries the CaMV 35S dual promoter-enhancer followed by the TEV leader and the CaMV 35S RNA polyadenylation signal sequences was used for construction of monocistronic and bicistronic vectors. This plasmid contains restriction sites for *Xho* I, *EcoR* I and *BamH* I and *Xba* I upstream and downstream of the TEV leader sequence, respectively (Figure 1). The genes encoding mGM-CSF and HBsAg were PCR-amplified using primers listed in Table 1 which contained *EcoR* I, *Xho* I and *BamH* I and *Bgl* II sites as required. All recombinant DNA manipulations were performed according to standard methods (Sambrook and Russell 2001).

**Table 1.** List of primers used for amplification of murine GM-CSF and HBsAg genes.

Primer	Sequence (5′–3′)	Product (bp)
F-GM	AGCATATGCCTCGAGGATCCATGGCCCACGAGAG	513
R-GM	TATACTATTCTCGAGGATCCCTATGCGGCCCC	
F-HBS	TTTTATAGATCTGAATTCATCTTCTCGAGGATTGGGGAC	783
R-HBS	TTTTTGGAATTCAGATCTAGAGTAACCCCATCTC	

mGM-CSF and HBsAg genes were amplified from pEF/myc/cyto/Δ7GM (kindly provided by Dr. H.J. Arteaga, Karolinska Institute, Stockholm, Sweden) and pCMV-S2S (a kind gift from Dr. Marie Louise Michel, Pasteur Institute, Paris, France), respectively. Amplification was performed at 94 °C for 5 min followed by 30 cycles of 94 °C for 1 min, 58 °C for 30 s and 72 °C for 1 min, and final extension at 72 °C for 10 min.

The amplified products were digested using either *EcoR* I (HBsAg gene) or *Xho* I (mGM-CSF gene) for upstream of TEV leader and *BamH* 1(mGM-CSF gene) or *Bgl* II (HBsAg gene) for downstream of the TEV leader in the configurations as shown in Figure 1. The plasmids carrying the expression cassette were sequenced (MWG Biotech AG, Ebersberg, Germany) for authentication of the PCR products and arrangement of the genes in the cassettes.

The modified pRTLs containing the expression cassettes were transformed to the Sure electroporation-competent *Escherichia coli* (Stratagen, Redmond, WA, USA) and transformants were selected on Luria Bertani (LB) agar plates containing 100 µg/mL ampicillin (Sigma, St. Louis, MO, USA). The expression cassettes were digested by *Hind* III, purified using gel extraction kit (Qiagen, Hilden, Germany) and ligated with *Hind* III digested binary vector pDE1001 (kindly provided by Dr. Ann Depicker, Ghent University, Belgium) (Denecke et al. 1992) as shown in schematic Figure 1.

After verification of the cloned pDE1001 vectors by sequencing, the recombinant vectors were used for transformation of electro-competent *Agrobacterium tumefaciens* C58CI Rif^R^ (a kind gift from Dr. G. De Jaeger, Ghent University, Belgium) using electroporation method.

### Agroinfiltration of tobacco leaves

Young leaves of *Nicotiana tabacum* cultivar Xanthi were agroinfiltrated (Kapila et al. 1997). Briefly, an isolated colony of transformed agrobacteria containing the cloned pDE1001 binary vectors was separately grown in 5 mL YEB medium supplemented with 50 µg/mL spectinomycin and 50 µg/mL rifampicin for 48 h at 28 °C in a shaker incubator (250 rpm). Subsequently, the 5 mL bacterial cultures of *Agrobacterium* harbouring the specific plasmid construct were used to separately inoculate 50 mL fresh YEB media containing 10 mM 2-(n-morpholino) ethane sulfonic acid (MES) (Merck, Darmstadt, Germany) adjusted pH to 5.6, and then 20 µM acetosyringone (Sigma, St. Louis, MO, USA) and the antibiotics were added to autoclaved media. The cultures were grown overnight at 28 °C to log phase (OD_600_=0.8 – 1.0) and centrifuged independently at 5000 rpm for 10 min at 4 °C. The pellets were resuspended in MMA medium containing Murashige and Skoog salts, 10 mM MES, 20 g/L sucrose, pH 5.6 and 200 µM acetosyringone to a final OD_600_ 2.4 and kept at 22 °C for 1 h and then used for infiltrations. Tobacco leaves were submerged in bacterial suspension and vacuumed at 0.1–1 mbar twice each for 10–15 min. Leaves must be kept in solution by shaking or heavy metallic material. The vacuum was broken rapidly each time to allow the agrobacteria-containing media to fill the interstitial spaces of the leaves replacing the vacuumed air. The infiltrated leaves were washed briefly with sterile water and kept on wet Whatman paper no. 40 with adaxial side upwards. Petri dishes were sealed with parafilm and placed at 22 °C under 16 h light/8 h dark condition.

### Preparation of agroinfiltrated plant leaf extract

After 3–5 days of incubation, the plant leaf extract was prepared. For the preparation, the leaves were frozen in liquid nitrogen and then crushed into fine powder and extracted with one volume of extraction buffer containing 20 mM sodium phosphate; pH 7.5, 0.15 M NaCl, 1% Triton X-100 and 2 mM PMSF. The samples were clarified by centrifugation at 17,000×*g* for 15 min at 4 °C. The supernatant was aliquoted and kept in freezer –80 °C for further experiments.

### SDS-PAGE and Western blot analysis

The samples were resuspended in sample buffer. The electrophoresis was done in 12% SDS PAG. After blotting, the nitrocellulose paper was put in BSA 3% overnight at 4 °C. The paper was put in 0.5 µg/mL biotinylated rabbit anti-GM-CSF antibody (Pharmingen, San Diego, CA, USA) for 2 h at 37 °C. After washing, the paper was incubated with 1:15,000 streptavidin conjugated peroxidase at room temperature for 1 h. The immunoblot was visualized using ECL Plus detection reagents (Amersham Pharmacia Biotech, Little Chalfont, UK) and developed on autoradiography film (Hyperfilm^TM^ ECL).

### HBsAg expression assays

Transient expression of HBsAg in agroinfiltrated leaves was measured using Monolisa kit according to manufacturer’s instruction (BioRad, Irvine, CA, USA) and sandwich ELISA. The test was done by monoclonal anti-HBsAg antibody 112A26 as coating and rabbit L3a_S2S antibody as secondary antibody (kindly donated by Dr. Marie-Louise Michel, Pasteur Institute, Paris, France). Goat affinity purified anti-rabbit IgG (H + L) peroxidase labelled antibodies (Vector Laboratories, Burlingame, CA) added at 1:3000 dilution in PBS.

### mGM-CSF expression assays

Transient expression of mGM-CSF was measured by sandwich ELISA assay. ELISA plates were coated with 1 µg/mL in PBS (pH 7.5) mouse anti-GM-CSF antibody (Pharmingen, San Diego, CA, USA) at 4 °C overnight. After three washes with PBS, the coated plates were blocked with 1% gelatin in TPBS (PBS containing 1% Tween-20) for 90 min at 37 °C. Plates were then washed and incubated with 100 µL of leaf extracts for 90 min at room temperature followed by three washes with TPBS. Then, 0.5 µg/mL biotinylated anti-GM-CSF was added to each well and further incubated for 90 min at room temperature followed by three washes with TPBS. Finally, 100 µL of 1:3000 dilution of streptavidin-peroxidase-conjugated goat anti-mouse antibody was added as the secondary antibody for 1 h. The plates were processed and developed by addition of OPD (1,2-phenylenediamine dihydrochloride; Dako, Santa Clara, CA, USA) and the reaction was stopped by 3 N HCl solution and colour development read at 492 nm.

### Tobacco plant extract cytotoxicity

Filter-sterilized soluble extract of tobacco leaves infiltrated by empty vector-engineered. *Agrobacterium* were passed through 22 µM filter and serially diluted to 1:128. The dilutions were incubated with 10^5^ cells of the immortalized mouse macrophage cell line J774A.1 overnight at 37 °C under 5% CO_2_. The cells were counted and cell viability (97%) was determined by Trypan blue exclusion dye. Minimum cytotoxicity was observed at the plant extract 1:32 dilution, so further experiments were done using the sterilized *Agrobacterium* infiltrated tobacco leaf soluble extract diluted to 1:40.

### Biological activity assay of mGM-CSF

BALB/c bone marrow cell suspensions were swiftly prepared from BALB/c mouse femurs (Lebastard et al. 1984), such bone marrow cell suspension being known to contain GM-CSF-responsive granulo-monocyte/GM progenitors. Different dilutions of the bone marrow cell suspensions were seeded in Terasaki plates in presence of soluble extract of tobacco leaves transformed with either control construct or with the mGM-CSF-engineered constructs and mouse rGM-CSF was used as positive control. Minimal frequency of GM progenitors was determined by limiting dilution analysis. The number of negative wells for each plated bone marrow cell dilution is counted. After the determination of negative wells percentage and its confidence limits the values were plotted on semi-log graph versus the number of cells plated. Assuming that the distribution of non-responding wells follows the Poisson distribution, the number of cells giving 37% negative wells contains a single progenitor (Lefkovits and Waldmann 1979).

## Results

### Expression cassettes’ construction

The amplified genes were digested by restriction enzymes and ligated into pRTL. The expression cassettes were sequenced and compared with original gene bank EMBL sequence using ClustalW multiples alignments program. The expression cassettes were introduced into TDNA of Binary vector pDEl00l (Figure 1) and the resulted vectors were introduced into electro-competent agrobacteria.

### Characterization of transient expressed recombinant proteins

**Figure 2.**
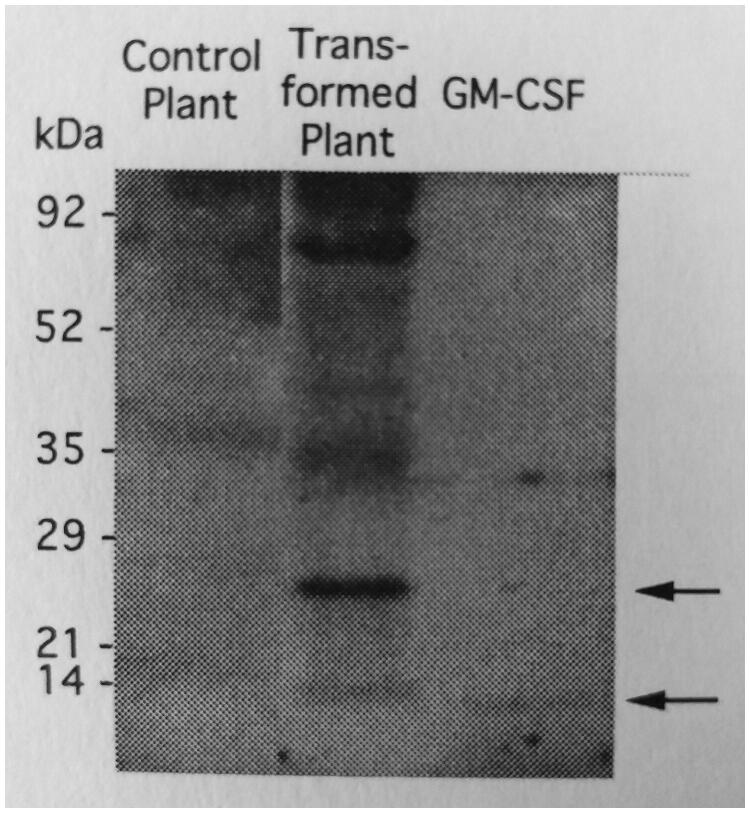
Western blot analysis of plant-produced mouse GM-CSF. Lane 1: tobacco leaves transformed by pDE1001 vector without mGM-CSF gene as negative control; lane 2: leaves were agroinfiltrated with the vector contained mGM-CSF gene; lane 3: *E. coli* derived GM-CSF.

The presence of the recombinant mGM-CSF and its molecular weight in agroinfiltrated tobacco leaves has been shown by western blotting (Figure 2). In the present study, the plant recombinant mGM-CSF has shown two specific bands; one is 27 kDa that corresponds to the glycosylated protein and other one corresponds to 14 kDa in un-glycosylated form. The results of ELISA tests indicated that the highest concentration of the plant-produced mGM-CSF is about 0.2–4 ng/mg of soluble proteins in tobacco leaf extract. In other ELISA assays for determining the HBsAg concentration, the expression level was varying from 20 to 50 ng/mg of tobacco leaf soluble protein.

### Frequency of GM-CSF-responsive progenitors in cell suspensions of mouse bone marrow by limiting dilution analysis

**Figure 3.**
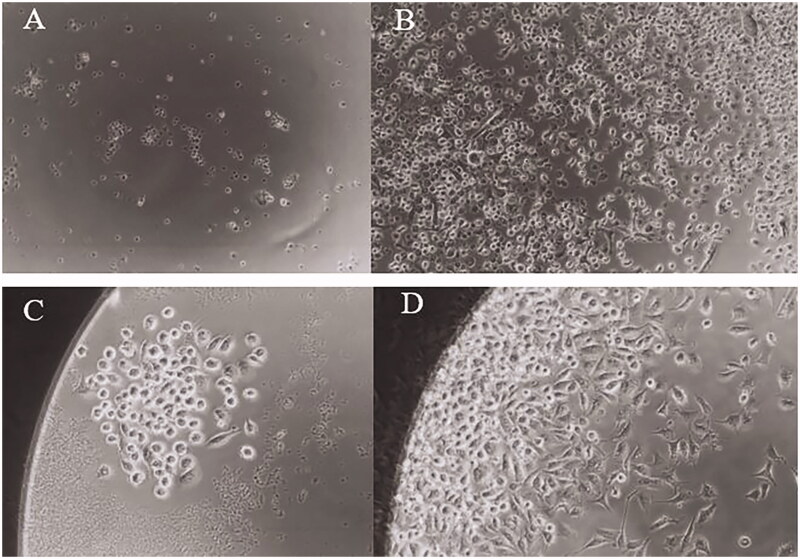
Identification of positive wells based on the ability of committed progenitor cells to form colonies of mature cells. A: empty vector plant extract as negative control; B: the bone marrow microcultures containing the plant produced mGM-CSF; C: the microplate containing the plant produced mGM-CSF; D: standard GM-CSF as positive control.

BALB/c mouse bone marrow cells were grown in microcultures in limiting dilution conditions containing transient expressed mGM-CSF plant extract or empty vector plant extract and standard GM-CSF. Following a growth period of seven days, the plates were scored for negative and positive wells. According to the Poisson distribution, the growth frequency of GM progenitors was approximately 1/187 cells for standard rGM-CSF and the plant produced mGM-CSF had 3.2 times less activity (Figure 3). The toxicity of the control plant extract for J774A.1 cells was diminished at 1:40 dilution.

### Analysis of the expression levels of mGM-CSF

**Figure 4.**
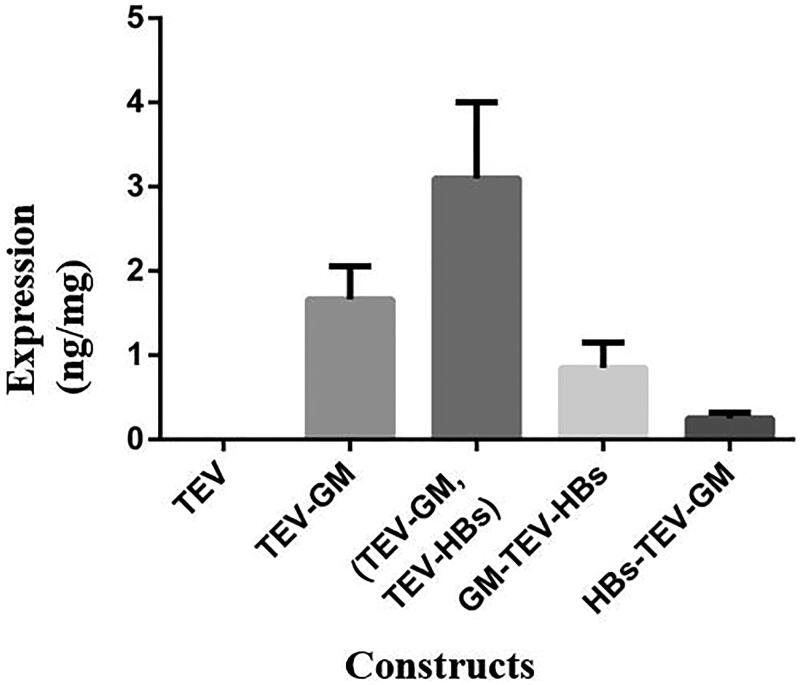
ELISA on plant extract to assess the expression level of GM-CSF in different bicistronic and monocistronic constructs. TEV indicates the leaves transformed by pDE1001 as negative control; TEV-GM, GM-TEV-HBs and HBs-TEV-GM are the leaves transformed by the monocistronic and bicistronic constructs, respectively (TEV-GM, TEV-HBs) indicates the leaves co-agroinfiltrated with both the GM-CSF and HBsAg monocistronic constructs.

The expression levels of mGM-CSF of different constructs were compared by ELISA (Figure 4). The leaves transformed by pDE1001 were used as negative control. The results indicated that the expression level of mGM-CSF was significantly higher in co-agroinfiltrated monocistronic constructs. It was followed by monocistronic mGM-CSF and bicistronic GM-TEV-HBS. While the least expression was seen in HBS-TEV-GM bicistronic construct carries mGM-CSF in second cistron and HBsAg gene in first cistron.

## Discussion

WHO’s emphasis on the mucosal and oral administration of vaccine and the potential of plant systems as host led to extensive studies on plant-based vaccine. Worldwide importance of hepatitis B infection with 350 million chronic carriers and almost 2 billion infected people has prompted scientists to search for a more applicable and effective vaccine. There are several publications on the capability of plant to produce precise conformation of HBsAg (Domansky et al. 1995; Mohammadzadeh et al. 2016). Furthermore, incorporation of adjuvants such as co-stimulatory portions in vaccine formulation is an effective strategy to break host immune tolerance and improve the immunogenicity of antigens especially in persistent infections or in oral administration rout. In oral delivery, plant expression systems are considered in bioencapsulation of biopharmaceuticals by the plant cell walls which provide a protecting layer during the digestive process in gastrointestinal tract (Criscuolo et al. 2019).

Beside HBsAg that has been shown to be correctly expressed and folded in transgenic plants, we tested transient co-expression of the functional bioactive mGM-CSF along with HBsAg by tobacco leaves because of its adjuvant importance to improve the immunological response against HBsAg in vaccine formulation.

The presence of mGM-CSF cytokine and HBsAg was checked by ELISA. The concentration of transient expressed HBsAg was varying from 20 to 50 ng/mg of soluble protein in the leaf extract. The expression level in different preparations was varied because there are several parameters that influence the expression level in transient expression such as competency of the leaves, position of the leaves in the agrobacteria solution, and the parameters involved in the preparation of *Agrobacterium* culture and infiltration condition. The highest expression level of mGM-CSF is about 4 ng/mg of leaf soluble protein which is higher in comparison to the expression level of human GM-CSF in optimized sterile suspension culture with 43 ng/g of callus (James et al. 2000). The low activity of the plant mGM-CSF (3.2-fold less compared to standard GM-CSF) (Figure 3(C)) in comparison to supernatant of rGM-CSF culture (Figure 3(D)) could be due to the presence of sub lethal concentration of some inhibitors in plant extract that interferes with normal metabolic pathways of progenitor cells or lower activity of plant produced recombinant cytokine that needed to be further investigated.

The molecular weight of the mGM-CSF in unglycosylated form is below 15 kDa. Due to post-translational glycosylation, the transient expressed mGM-CSF in tobacco leaves observed in western blot analysis is 27 kDa (Figure 2) that corresponds to glycosylated form of mouse GM-CSF purified 23 kDa glycoprotein (Nicola et al. 1979). The mouse GM-CSF protein produced by plant cells has been reported to be glycosylated and the possibility of N-glycosylation on *in silico* predicted sites (positions 66 and 75) was verified (Góra-Sochacka et al. 2010; Liu et al. 2012). As shown in Figure 3, the growth of GM-CSF-reactive progenitors cells was actively stimulated by the plant produced mGM-CSF. *In vitro* analysis of the biological activity has already shown that nonglycosylated and glycosylated forms of the plant produced mouse GM-CSF have the same activity (Góra-Sochacka et al. 2010). However, the glycosylation is essential to increase *in vivo* stability of GM-CSF that could vary according to host cell type (Kim et al. 2008).

In order to be able to co-express both the antigen and costimulatory cytokine in the plant cell in non-fusion form, we evaluated the monocistronic and bicistronic expression cassettes in the presence of noncoding TEV leader sequences (Figure 1). The comparison of the mGM-CSF expression levels indicated that inserting TEV leader sequence in the upstream of the mGM-CSF gene in TEV-GM construct increased the expression level significantly compared to GM-TEV-HBs construct that TEV placed in the downstream of the gene (Figure 4). This finding is based on the study by James et al. (2000) showing that the addition of the TEV leader sequence upstream of the human GM-CSF transgene increased the protein production more than twofold compared to non-TEV controls due to translational efficiency enhancement (James et al. 2000). The lower level of mGM-CSF expression in plants received HBs-TEV-GM bicistronic construct (Figure 4) may be due to the formation of a strong stem-loop structure at the end of HBsAg mRNA that is located in the upstream of the TEV leader. Niepel and Gallie (1999) have shown that the formation of the stable stem-loop structure led to the blockage of ribosomal scanning and decreased its translational ability significantly (Niepel and Gallie 1999). Therefore, an accessible 5′ end for optimal translation is required and should be considered.

The ELISA results indicated that the greatest mGM-CSF expression level was gained in plants co-agroinfiltrated with both HBsAg and mGM-CSF monocistronic constructs having promoter and TEV leader (Figure 4). It was 1.5-fold higher than the amount of mGM-CSF produced in plants received monocistronic construct solely. It seems that the two constructs containing TEV sequence have synergistic effect on the ribosomal recruitment and enhancement of mGM-CSF translation.

Therefore, transient expression systems show several applications in research such as comparative analysis of plant promoters and regulatory elements, quick production of protein of interest for verification of the stability and functionality of plant produced recombinant proteins. Moreover, it is deduced that the secondary structure of the mRNA of the whole construct affects the second cistron using TEV-based cassettes.

## References

[CIT0001] Carrington JC, Freed DD. 1990. Cap-independent enhancement of translation by a plant potyvirus 5′ nontranslated region. J Virol. 64(4):1590–1597. 2319646 10.1128/jvi.64.4.1590-1597.1990 PMC249294

[CIT0002] Catherine F-X, Piroth L. 2017. Hepatitis B virus vaccination in HIV-infected people: a review. Hum Vaccin Immunother. 13(6):1–1313. 10.1080/21645515.2016.1277844 PMC5489285 28267387

[CIT0003] Chou HY, Lin XZ, Pan WY, Wu PY, Chang CM, Lin TY, Shen HH, Tao MH. 2010. Hydrogel-delivered GM-CSF overcomes nonresponsiveness to hepatitis B vaccine through the recruitment and activation of dendritic cells. J Immunol. 185(9):5468–5475. 20889541 10.4049/jimmunol.1001875

[CIT0004] Criscuolo E, Caputo V, Diotti R, Sautto G, Kirchenbaum G, Clementi N. 2019. Alternative methods of vaccine delivery: an overview of edible and intradermal vaccines. J Immunol Res. 2019:13. 10.1155/2019/8303648 PMC6425294 30949518

[CIT0005] Decker WK, Safdar A. 2011. Cytokine adjuvants for vaccine therapy of neoplastic and infectious disease. Cytokine Growth Factor Rev. 22(4):177–187. 21862380 10.1016/j.cytogfr.2011.07.001

[CIT0006] Denecke J, De Rycke R, Botterman J. 1992. Plant and mammalian sorting signals for protein retention in the endoplasmic reticulum contain a conserved epitope. EMBO J. 11(6):2345–2355. 1376250 10.1002/j.1460-2075.1992.tb05294.x PMC556702

[CIT0007] Domansky N, Ehsani P, Salmanian A-H, Medvedeva T. 1995. Organ-specific expression of hepatitis B surface antigen in potato. Biotechnol Lett. 17(8):863–866.

[CIT0008] Ehsani P, Khabiri A, Domansky NN. 1997. Polypeptides of hepatitis B surface antigen produced in transgenic potato. Gene. 190(1):107–111. 9185855 10.1016/s0378-1119(96)00647-6

[CIT0009] Góra-Sochacka A, Redkiewicz P, Napiórkowska B, Gaganidze D, Brodzik R, Sirko A. 2010. Recombinant mouse granulocyte-macrophage colony-stimulating factor is glycosylated in transgenic tobacco and maintains its biological activity. J Interferon Cytokine Res. 30(3):135–142. 20038209 10.1089/jir.2009.0053

[CIT0010] James EA, Wang C, Wang Z, Reeves R, Shin JH, Magnuson NS, Lee JM. 2000. Production and characterization of biologically active human GM-CSF secreted by genetically modified plant cells. Protein Expr Purif. 19(1):131–138. 10833400 10.1006/prep.2000.1232

[CIT0011] Joung Y, Park S, Moon K-B, Jeon J-H, Cho H-S, Kim H-S. 2016. The last ten years of advancements in plant-derived recombinant vaccines against hepatitis B. Int J Mol Sci. 17(10):1715. 10.3390/ijms17101715 PMC5085746 27754367

[CIT0012] Kapila J, De Rycke R, Van Montagu M, Angenon G. 1997. An agrobacterium-mediated transient gene expression system for intact leaves. Plant Sci. 122(1):101–108.

[CIT0013] Kim TG, Lee HJ, Jang YS, Shin YJ, Kwon TH, Yang MS. 2008. Co-expression of proteinase inhibitor enhances recombinant human granulocyte-macrophage colony stimulating factor production in transgenic rice cell suspension culture. Protein Expr Purif. 61(2):117–121. 18634882 10.1016/j.pep.2008.06.005

[CIT0014] Kopertekh L, Schiemann J. 2019. Transient production of recombinant pharmaceutical proteins in plants: evolution and perspectives. Curr Med Chem. 26(3):365–380. 28721831 10.2174/0929867324666170718114724

[CIT0015] Lebastard M, Milon G, Marchal G. 1984. A new assay suitable for enumeration of murine progenitors of granulo-monocytes and for rapid automated assessment of granulo-monocyte growth factors. J Immunol Methods. 67(1):173–183. 6607957 10.1016/0022-1759(84)90096-6

[CIT0016] Lefkovits I, Waldmann H. 1979. Limiting dilution analysis of cells in the immune system. Cambridge, UK: Cambridge University Press.

[CIT0017] Li X, Liu X, Tian L, Chen Y. 2016. Cytokine-mediated immunopathogenesis of hepatitis B virus infections. Clin Rev Allergy Immunol. 50(1):41–54. 25480494 10.1007/s12016-014-8465-4

[CIT0018] Lin C, Zhu J, Zheng Y, Chen Y, Wu Z, Chong Y, Gao Z. 2010. Effect of GM-CSF in combination with hepatitis B vaccine on revaccination of healthy adult non-responders. J Infect. 60(4):264–270. 20138189 10.1016/j.jinf.2010.01.011

[CIT0019] Liu YK, Huang LF, Ho SL, Liao CY, Liu HY, Lai YH, Yu SM, Lu CA. 2012. Production of mouse granulocyte-macrophage colony-stimulating factor by gateway technology and transgenic rice cell culture. Biotechnol Bioeng. 109(5):1239–1247. 22125231 10.1002/bit.24394

[CIT0020] Margolin E, Chapman R, Williamson AL, Rybicki EP, Meyers AE. 2018. Production of complex viral glycoproteins in plants as vaccine immunogens. Plant Biotechnol J. 16(9):1531–1545. 10.1111/pbi.12963 PMC6097131 29890031

[CIT0021] Mohammadzadeh S, Rahimi S, Ebrahimi-Rad M, Ofoghi H, Ehsani P. 2017. Transient expression of virus-like particles in plants: a promising platform for rapid vaccine production. Vac Res. 4:72–80.

[CIT0022] Mohammadzadeh S, Roohvand F, Memarnejadian A, Jafari A, Ajdary S, Salmanian AH, Ehsani P. 2016. Co-expression of hepatitis C virus polytope-HBsAg and p19-silencing suppressor protein in tobacco leaves. Pharm Biol. 54(3):465–473. 25990925 10.3109/13880209.2015.1048371

[CIT0023] Nicola N, Metcalf D, Johnson G, Burgess A. 1979. Separation of functionally distinct human granulocyte-macrophage colony-stimulating factors. Blood. 54(3):614–627. 313822

[CIT0024] Niepel M, Gallie DR. 1999. Identification and characterization of the functional elements within the tobacco etch virus 5′ leader required for cap-independent translation. J Virol. 73(11):9080–9088. 10516014 10.1128/jvi.73.11.9080-9088.1999 PMC112940

[CIT0025] Ofoghi H, Moazami N, Domonsky N, Ivanov I. 2000. Cloning and expression of human calcitonin genes in transgenic potato plants. Biotechnol Lett. 22(7):611–615.

[CIT0026] Pniewski T, Milczarek M, Wojas-Turek J, Pajtasz-Piasecka E, Wietrzyk J, Czyż M. 2018. Plant lyophilisate carrying S-HBsAg as an oral booster vaccine against HBV. Vaccine. 36(41):6070–6076. 30197284 10.1016/j.vaccine.2018.09.006

[CIT0027] Qing Y, Chen M, Zhao J, Hu H, Xu H, Ling N, Peng M, Ren H. 2010. Construction of an HBV DNA vaccine by fusion of the GM-CSF gene to the HBV-S gene and examination of its immune effects in normal and HBV-transgenic mice. Vaccine. 28(26):4301–4307. 20430121 10.1016/j.vaccine.2010.04.023

[CIT0028] Roozbeh J, Bagheri-Lankarani K, Mohaghegh P, Raeesjalali G, Behzadi S, Sagheb M, Vossoughi M, Bastani B. 2015. A randomized pilot trial on the effect of granulocyte-colony stimulating factor on antibody response in hemodialysis patients who had not responded to routine hepatitis B virus vaccine. J Nephropathol. 4(1):13–17. 25657980 10.12860/jnp.2015.03 PMC4316580

[CIT0029] Sambrook J, Russell DW. 2001. Molecular cloning: a laboratory manual. New York: Cold Spring Harbor Laboratory Press.

[CIT0030] Sardana RK, Alli Z, Dudani A, Tackaberry E, Panahi M, Narayanan M, Ganz P, Altosaar I. 2002. Biological activity of human granulocyte-macrophage colony stimulating factor is maintained in a fusion with seed glutelin peptide. Transgenic Res. 11(5):521–531. 12437083 10.1023/a:1020343501475

[CIT0031] Sasaki MG, Foccacia R, de Messias-Reason IJ. 2003. Efficacy of granulocyte-macrophage colony-stimulating factor (GM-CSF) as a vaccine adjuvant for hepatitis B virus in patients with HIV infection. Vaccine. 21(31):4545–4549. 14575766 10.1016/s0264-410x(03)00500-0

[CIT0032] Shin YJ, Hong SY, Kwon TH, Jang YS, Yang MS. 2003. High level of expression of recombinant human granulocyte-macrophage colony stimulating factor in transgenic rice cell suspension culture . Biotechnol Bioeng. 82(7):778–783. 12701143 10.1002/bit.10635

[CIT0033] Vojta L, Ljuma-Skupnjak L, Budimir A, Vukičević S, Fulgosi H. 2015. Rapid transient expression of human granulocyte-macrophage colony-stimulating factor in two industrial cultivars of tobacco (*Nicotiana tabacum* L.) by agroinfiltration. Biotechnol Rep (Amst). 7:81–86. 28626718 10.1016/j.btre.2015.05.006 PMC5466047

[CIT0034] Zeenko V, Gallie DR. 2005. Cap-independent translation of tobacco etch virus is conferred by an RNA pseudoknot in the 5′-leader. J Biol Chem. 280(29):26813–26824. 15911616 10.1074/jbc.M503576200

[CIT0035] Zhou F, Wang M-L, Albert HH, Moore PH, Zhu YJ. 2006. Efficient transient expression of human GM-CSF protein in *Nicotiana benthamiana* using potato virus X vector. Appl Microbiol Biotechnol. 72(4):756–762. 16612640 10.1007/s00253-005-0305-2

